# Supplementary material for: Resolving the full spectrum of human genome variation using Linked-Reads
Source: Genome Res. 2019 Apr;29(4):635–45. doi: 10.1101/gr.234443.118 (PMC6442396; doi:10.1101/gr.234443.118)
Supplement: Supplemental Material [file supp_gr.234443.118_Supplemental_longranger-2.2.zip › longranger-2.2.tar.bz2!/lr2.2-public/lib/stan/cmdstan/stan/doc/api/html/classstan_1_1common_1_1recorder_1_1no__op.html]

Stan: stan::common::recorder::no\_op Class Reference


|  |  |
| --- | --- |
|  | Stan  2.5.0  probability, sampling & optimization |


- Main Page
- Related Pages
- Namespaces
- Classes
- Files

- Class List
- Class Index
- Class Hierarchy
- Class Members

All Classes Namespaces Files Functions Variables Typedefs Enumerations Enumerator Friends Macros Pages

- stan
- common
- recorder
- no\_op

Public Member Functions |
List of all members

stan::common::recorder::no\_op Class Reference

Does nothing.
More...

`#include <no_op.hpp>`

|  |  |
| --- | --- |
| Public Member Functions | |
| template<class T > | |
| void | operator() (const std::vector< T > &x) |
|  | Do nothing with vector. More... |
|  | |
| void | operator() (const std::string x) |
|  | Do nothing with a string. More... |
|  | |
| void | operator() () |
|  | Do nothing. More... |
|  | |
| bool | is\_recording () const |
|  | Indicator function for whether the instance is recording. More... |
|  | |

## Detailed Description

Does nothing.

Definition at line 15 of file no\_op.hpp.

## Member Function Documentation

|  |  |  |  |  |
| --- | --- | --- | --- | --- |
| bool stan::common::recorder::no\_op::is\_recording | ( |  | ) | const |

inline

Indicator function for whether the instance is recording.

Definition at line 42 of file no\_op.hpp.

template<class T >

|  |  |  |  |  |  |
| --- | --- | --- | --- | --- | --- |
| void stan::common::recorder::no\_op::operator() | ( | const std::vector< T > & | *x* | ) |  |

inline

Do nothing with vector.

Template Parameters
:   |  |  |
    | --- | --- |
    | T | type of element |

Parameters
:   |  |  |
    | --- | --- |
    | x | vector of type T |

Definition at line 24 of file no\_op.hpp.

|  |  |  |  |  |  |
| --- | --- | --- | --- | --- | --- |
| void stan::common::recorder::no\_op::operator() | ( | const std::string | *x* | ) |  |

inline

Do nothing with a string.

Parameters
:   |  |  |
    | --- | --- |
    | x | string to print with prefix in front |

Definition at line 31 of file no\_op.hpp.

|  |  |  |  |  |
| --- | --- | --- | --- | --- |
| void stan::common::recorder::no\_op::operator() | ( |  | ) |  |

inline

Do nothing.

Definition at line 37 of file no\_op.hpp.

---

The documentation for this class was generated from the following file:

- src/stan/common/recorder/no\_op.hpp


---

[ Stan Home Page ]

*© 2011–2014,
Stan Development Team.*
